# Evolutionary and Functional Diversity of the 5′ Untranslated Region of Enterovirus D68: Increased Activity of the Internal Ribosome Entry Site of Viral Strains during the 2010s

**DOI:** 10.3390/v11070626

**Published:** 2019-07-08

**Authors:** Yuki Furuse, Natthawan Chaimongkol, Michiko Okamoto, Hitoshi Oshitani

**Affiliations:** 1 Institute for Frontier Life and Medical Sciences, Kyoto University, 53 Shogoin Kawaracho, Sakyo-ku, Kyoto 606-8507, Japan; 2 Hakubi Center for Advanced Research, Kyoto University, Yoshidahonmachi, Sakyo-ku, Kyoto 606-8501, Japan; 3 Department of Virology, Tohoku University Graduate School of Medicine, 2-1 Seiryomachi, Aoba-ku, Sendai 980-8575, Japan; 4 Frontier Research Institute for Interdisciplinary Sciences, Tohoku University, 6-3 Aramaki aza Aoba, Aoba-ku, Sendai 980-8578, Japan

**Keywords:** enterovirus, evolution, noncoding region, untranslated region, internal ribosome entry site, phylogeny

## Abstract

The 5′ untranslated region (UTR) of the RNA genomes of enteroviruses possesses an internal ribosome entry site (IRES) that directs translation of the mRNA by binding to ribosomes. Infection with enterovirus D68 causes respiratory symptoms and is sometimes associated with neurological disorders. The number of reports of the viral infection and neurological disorders has increased in 2010s, although the reason behind this phenomenon remains unelucidated. In this study, we investigated the evolutionary and functional diversity of the 5′ UTR of recently circulating strains of the virus. Genomic sequences of 374 viral strains were acquired and subjected to phylogenetic analysis. The IRES activity of the viruses was measured using a luciferase reporter assay. We found a highly conserved sequence in the 5′ UTR and also identified the location of variable sites in the predicted RNA secondary structure. IRES activities differed among the strains in some cell lines, including neuronal and respiratory cells, and were especially high in strains of a major lineage from the recent surge. The effect of mutations in the 5′ UTR should be studied further in the future for better understanding of viral pathogenesis.

## 1. Introduction

Enterovirus D68 (EV-D68), which belongs to the family *Picornaviridae*, has a single-stranded positive-sense RNA genome, that is ~7,400 bases in length and encodes a single polyprotein [1]. Virus-coded proteases cleave the polyprotein into several structural and nonstructural viral proteins. The virion is non-enveloped, and the capsid is composed of four structural subunits: VP1, VP2, VP3, and VP4 [2] and antibodies against VP1 neutralize viral infectivity [3,4]. There is an untranslated region (UTR) at both ends of the enterovirus genome, which does not have a 5′ cap, although its 5′ end is covalently linked to a viral protein named VPg. The highly structured 5′ UTR of the RNA genome of the virus contains an internal ribosome entry site (IRES) that directs translation of viral mRNA by binding to ribosomes [5]. Enteroviruses possess a type I IRES that consists of 6 (I–VI) stem-loop structures [6,7]. Translation initiation via a type I IRES involves binding to the 40S ribosomal subunit through interaction with eIF3, eIF4A, and eIF4G [6,7]. The IRES plays a role in virulence, since mutations in this region in enteroviruses, including poliovirus, are reported to affect their virulence and neural tropism [8,9,10].

EV-D68 was first isolated from children with respiratory symptoms in 1962 and there have been sporadic reports of outbreaks since then [11]. Infection causes severe respiratory symptoms in some patients and is sometimes associated with neurological disorders, such as acute flaccid paralysis [12]. Outbreaks of the viral infection have occurred worldwide between 2014 and 2016 [12], and there should be biological and/or epidemiological reasons behind the recent global surge of this virus. We previously reported that recently circulating strains of the virus differ antigenically from a prototype virus (Fermon/1962) and possess a genomic signature in the VP1 gene [4,13], and yet, the exact cause of the recent viral outbreaks remains unknown. In addition, while the number of reports of neurological disorders from infection with EV-D68 has been increasing, it remains unknown whether recently circulating strains are more virulent than older strains, or whether the large scale of the outbreaks has simply made such cases more evident [12,14,15].

Since genetic diversity in the 5′ UTR of recently circulating strains of EV-D68 has been reported [14,16], it is possible that the 5′ UTR in the viral genome has evolved and produced physiological differences among strains that could have played roles in the recent surge of the viral infection and/or its neurovirulence. In this study, we analyzed evolutionary and functional diversity in the 5′ UTR of this virus.

## 2. Materials and Methods

### 2.1. Genomic Data

All available sequence data of the whole genome of EV-D68 (*N* = 442) were obtained from GenBank (accessed on 2 May 2017). After data cleaning and alignment of the sequences, 374 viral strains, collected between 1962 and 2016, and with sequences containing at least the 7218-nucleotide region (corresponding to positions 33–7299 of Fermon/1962), were selected for further analysis. Data of genomic sequence of 374 strains, analyzed in the study, are available in Supplementary Data S1.

### 2.2. Phylogenetic Analysis

Pairwise genetic distances along whole genomes, among the 374 strains, were determined using a sliding window of 100 nucleotides with a step size of 10 nucleotides and were calculated using SSE version 1.3 [17]. Phylogenetic trees for the 5′ UTR and VP1 gene of the 374 strains were constructed with bootstrap values from 500 iterations, using the maximum likelihood method, based on the general time-reversible model using MEGA7 [18]. A discrete gamma distribution was used to model evolutionary rate differences between sites, and the rate variation model allowed for some sites to be evolutionarily invariable.

### 2.3. Secondary Structure

The RNA secondary structure for each stem-loop structure of the 5′ UTR of EV-D68 (Fermon/1962) was predicted using RNA structure software [19] by referring to coxsackievirus B3 [6,7]. The locations of variable sites in the predicted structure were determined in the consensus sequence of each phylogenetic lineage by comparison with the prototype (Fermon/1962).

### 2.4. Cell Lines

HEK293T, HEp2, and RD cells were maintained in DMEM supplemented with 10% fetal bovine serum (FBS), A172 cells were maintained in RPMI 1640, supplemented with 10% FBS, and HFL cells were maintained in DMEM/Ham’s F12 medium, supplemented with 10% FBS. All cell lines were obtained from RIKEN BioResource Research Center (Tsukuba, Japan).

### 2.5. Viruses

Our archived viral strains were isolated from clinical nasopharyngeal specimens from pediatric patients with respiratory symptoms in Japan and the Philippines, that were collected during our previous studies [13,20,21,22]. Reverse transcription-polymerase chain reaction (RT-PCR) was conducted after nucleic acid extraction from the viral isolates, using a QIAamp MinElute Virus Spin kit (Qiagen, Hilden, Germany) [21]. The PCR amplicon was then sequenced using an ABI 3730xl with BigDye version 1.1 (Thermo Fisher Scientific, Waltham, MA, USA) to determine the nucleotide sequence of the 5′ UTR and VP1 of the viral strains, and find their phylogenetic lineage. Viral strains named Y2167/2010 and Y2071/2010 (Lineage 1), Ph364/2013 and Ph397/2013 (Lineage 2), and Ph224/2011 and Y2256/2010 (Lineage 3), were used for cloning to represent each phylogenetic lineage. Genetic sequence of the 5′ UTR of the six strains was available at GenBank (Accession number: LC477344–LC477349).

### 2.6. Plasmids

RT-PCR amplified DNAs, corresponding to the entire region of the 5′ UTR of EV-D68, Fermon/1962 (ATCC, Manassas, VA, USA), and viral strains from our archived samples were sub-cloned into a bicistronic plasmid, which contained a cytomegalovirus promoter, the Renilla luciferase (Rluc) gene, a cloning site, the firefly luciferase (Fluc) gene, and a bovine growth hormone polyadenylation signal. A chimeric DNA sequence of the 5′ UTR of Fermon/1962 and Ph397/2013 was also synthesized (Integrated DNA technologies, Coralville, IA, USA) and subcloned into the plasmid.

### 2.7. Luciferase Assay

One hundred ng of each plasmid was transfected into cells of each line seeded into 96-well plates at ~60% confluency using jetPRIME (Polyplus-transfection, Illkirch, France). Cells were lysed 24 h after transfection, and Fluc and Rluc activities were measured using a Dual-Glo Luciferase Assay System (Promega, Madison, WI, USA). Experiments were conducted in triplicate.

### 2.8. Viral Growth Assay

Seed viral strains of Fermon/1962 and Ph397/2013 were propagated in RD cells. The viruses were then used to infect A172 cells at a multiplicity of infection (MOI) of 0.01. Culture supernatants and cellular lysates were collected at 12, 24 and 48 h post-infection. Viral RNA levels in these samples were measured by quantitative RT-PCR using previously described primer [23] and StepOnePlus (Thermo Fisher Scientific, Waltham, MA, USA). Progeny virus in the supernatants was titrated using RD cells.

### 2.9. Statistical Analysis

One-way analysis of variance with Dunnett post hoc test was performed for Fluc activities normalized with Rluc activities, in order to compare IRES activities among the 5′ UTRs of recently circulating strains with Fermon/1962. The same test was performed to compare IRES activities of the 5′ UTRs of the chimeric sequences of Fermon/1962 and Ph397/2013 (lineage 2) with their parental sequence. The student’s *t*-tests were performed to compare viral titer and viral RNA levels in growth assays between Fermon/1962 and Ph397/2013. P-values below 0.05 were considered statistically significant. All statistical tests were performed using SPSS ver. 24 (IBM, Armonk, NY, USA).

## 3. Results

**Figure 1:**
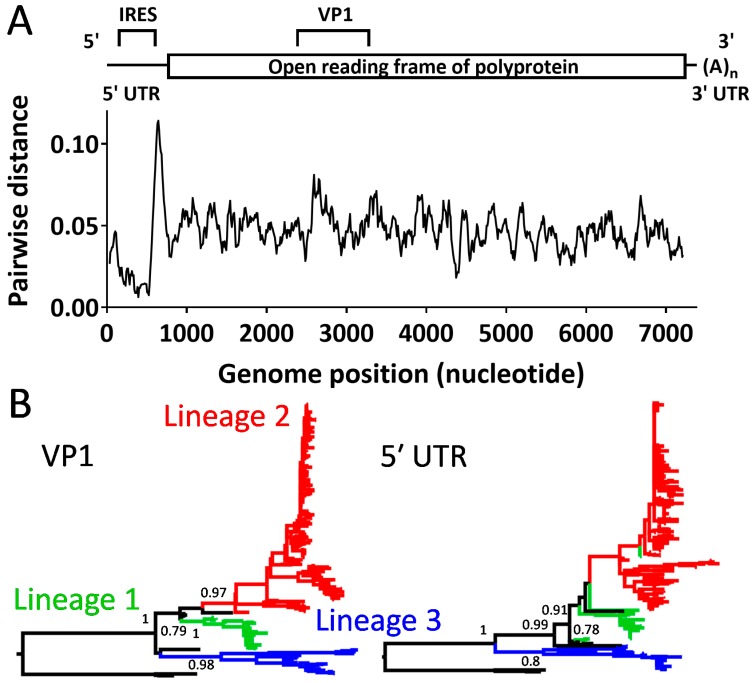
Genetic diversity of the 5′ UTR of EV-D68 (**A**) Average pairwise genetic diversity of the whole genome of EV-D68 among the 374 strains collected between 1962 and 2016, calculated using a sliding window of 100 nucleotides with a step size of 10 nucleotides. Schematic figure for the structure of whole genome is shown at the top. (**B**) Phylogenetic trees of the viral strains, constructed using the maximum likelihood method for the VP1 gene (left) and the 5′ UTR (right). Branches are colored by lineages identified by the phylogenetic tree for the VP1 gene. Bootstrap values >60% at major branches are shown.

Pairwise genetic diversity along the EV-D68 genome showed that the diversity among the 374 strains was highest at the 3′ end of the 5′ UTR (i.e., the 5′ region upstream of the open reading frame of the polyprotein) and lowest at the IRES region within the 5′ UTR (Figure 1A). Genetic diversity in a region encoding VP1 that determines antigenicity was relatively high compared with other regions of the genome. Recently circulating strains of EV-D68 were classified into three lineages based on the nucleotide sequence of the VP1 region, namely, lineages 1 (clade C), 2 (clade B), and 3 (clade A) [20,24]. The phylogenetic analysis showed that the three lineages are also distinct from each other for the 5′ UTR sequence (Figure 1B). These results suggested that there are lineage-specific mutations in the 5′ UTR, although each strain had a few variations even within the same lineage. Interestingly, the 5′ UTR sequence of a lineage 1 strain, USA/O622a/2012, was clustered with lineage 2 strains. The strain was found to be a recombinant between 5′UTR of lineage 2 and the open reading frame of the polyprotein of lineage 1.

**Figure 2:**
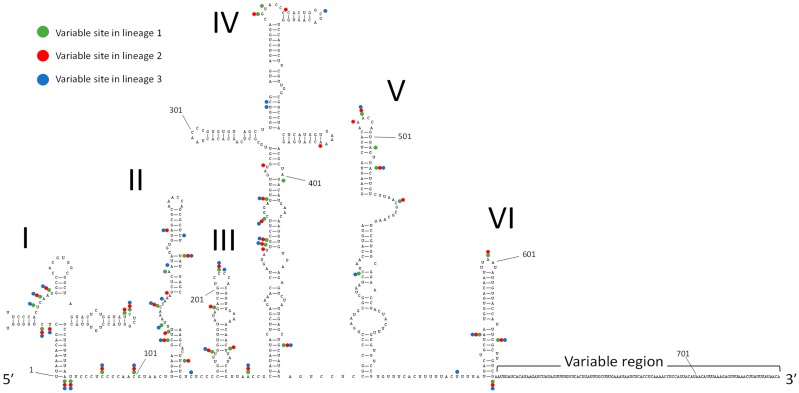
RNA secondary structure of the 5′ UTR of EV-D68. Predicted RNA secondary structure of the 5′ UTR of EV-D68 (Fermon/1962) is shown. Locations of mutations in the consensus sequence of viral strains in each phylogenetic lineage are indicated by dots except variable region. The Green, blue, and red indicate mutation sites in lineages 1, 2, and 3, respectively. The same figure in high resolution is available in Figure S1.

Figure 2 illustrates the predicted secondary structure of the 5′ UTR of EV-D68 (Fermon/1962). The structure is typical of a type I IRES and has six stem-loops (I–VI) [6,7]. Mutated sites in the 5′ UTR of recently circulating strains in each lineage, using consensus sequences, were compared with Fermon/1962, and their locations in the secondary structure were identified (Figure 2). Genetic variations of recently circulating strains were located throughout the IRES structure. Interestingly, many of the mutations in the stem structure would maintain base-pairing (e.g., mutations from A–U to G–U pairs). In addition, while Fermon/1962 has 108 nucleotides in the variable region of the 5′ UTR, the corresponding regions of the recently circulating strains were shorter, ranging between 71 and 86 nucleotides.

**Figure 3:**
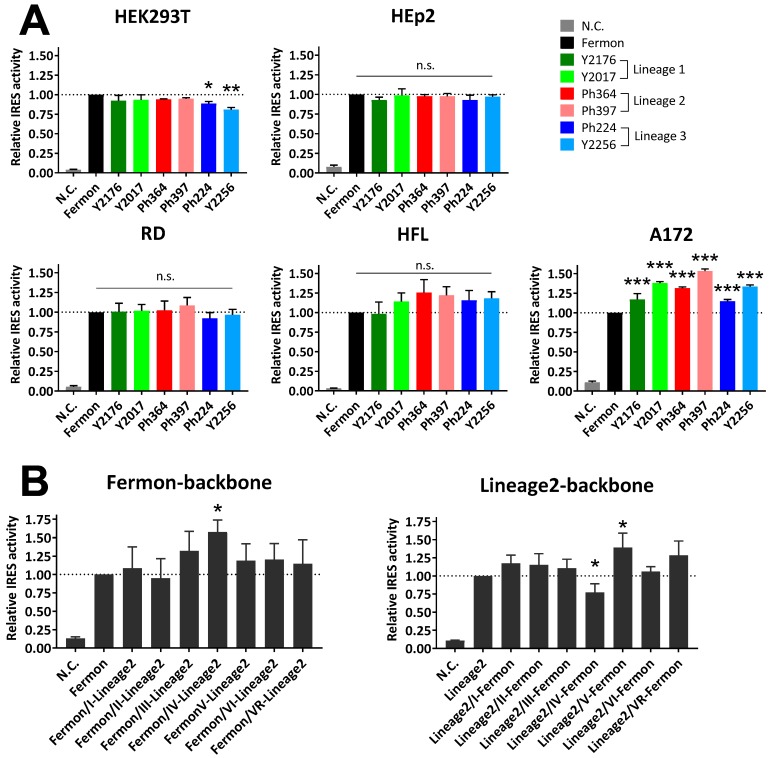
IRES activities of the 5′ UTR of EV-D68 in various cell lines. (**A**) IRES activities of the 5′ UTR of Fermon/1962 and recently circulating strains of EV-D68 as determined in various cell lines. The activities were measured by luciferase activity and normalized to Fermon/1962. N.C. indicates a negative control, which was a construct lacking the 5′ UTR of EV-D68. (**B**) IRES activities of 5′ UTR of chimeric sequences of Fermon/1962 and lineage 2 strain (Ph397/2013) in A172 cells. The RNA sequence in each stem-loop (I–VI) or variable region (VR) was exchanged between the two strains. The activities were measured by luciferase activity and normalized to the backbone sequence. Data represent the mean ± standard deviation of three independent experiments. *, *P* < 0.05; **, *P* < 0.01; ***, *P* < 0.001; n.s., not significant.

We then checked whether the genetic variation in the 5′ UTR affected IRES activity. Bicistronic plasmids, that contain a reporter gene (Fluc) under the control of the 5′ UTRs from the different strains of EV-D68, were constructed to measure their IRES activities in different cell lines (Figure 3A). We used the 5′ UTR sequence of 2 strains from our archived samples for each phylogenetic lineage. There were no significant differences in IRES activities between the prototype (Fermon/1962) and the recently circulating strains in HEp2 and RD cells, which are widely used for virus isolation. In contrast, the IRES activities of the recently circulating strains were slightly higher than that of the prototype in A172 neuronal glioblastoma cells (with statistical significance) and HFL lung fibroblast cells (without statistical significance), especially for lineage 2 strain.

**Figure 4:**
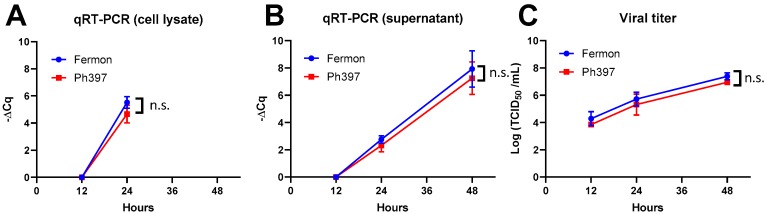
Growth kinetics of Fermon/1962 and Ph397/2013 (lineage 2) in A172 cells. A172 cells were infected with a virus at a MOI of 0.01. Supernatant and cellular lysates were collected at 12, 24, and 48 h after infection. Viral RNA levels in cellular lysates (**A**) and supernatants (**B**) were measured by quantitative RT-PCR and shown as differences in Cq values, compared with data at 12 h. Data for cellular lysates at 48 h were unavailable because of cellular death, due to the cytopathic effect. Progeny viruses in the supernatants were titrated using the 50% tissue culture infective dose (TCID_50_) method in RD cells. (**C**) Data represent the mean ± standard deviation of three independent experiments. n.s., not significant.

Notably, the IRES activity of the 5′ UTR of Ph397/2013 (lineage 2) in A172 cells was significantly higher than that of, not only Fermon/1962, but also the other recently circulating strains. We therefore further analyzed the lineage 2 strain generating a chimeric sequence of the 5′ UTRs of Fermon/1962 and Ph397/2013 to determine the region responsible for the increased IRES activity in A172 cells. The chimeric sequence, consisting of the Fermon/1962-backbone with the lineage 2 stem-loop IV, exhibited statistically higher IRES activity than the parental sequence, whereas the chimeric sequence, consisting of the lineage 2-backbone with Fermon/1962 stem-loop IV, possessed statistically lower IRES activity than its parental sequence (Figure 3B). Finally, we checked the viral growth kinetics of the two viral strains, Fermon/1962 and Ph397/2013 (lineage 2), in A172 cells but found no statistical difference between them (Figure 4).

## 4. Discussion

In this study, we described (1) a genetic diversity in nucleotide sequence of the 5′ UTR of the genome of EV-D68; (2) the predicted RNA secondary structure of the 5′ UTR of the virus and the position of mutations observed in recently circulating strains; and (3) the differential cell line-dependent IRES activities among viral strains. While the IRES structure in the 5′ UTR was well conserved, there were a few mutations, some of which would affect IRES activity. It is interesting that the differences in IRES activities among the strains depended on which cell lines were used for testing, and these differences were not seen in cell lines that are widely used for virus isolation, namely, HEp2 and RD.

Strains in lineage 2 (clade B) were mainly responsible for the recent worldwide spread of this virus [13,14,25]. Although it is unclear whether the virus evolved increasing neurovirulence [12], an association between the recently circulating strains, including the ones in lineage 2, and neuronal disorders, has been reported both epidemiologically and experimentally [14,15,25,26,27,28,29,30]. The present study demonstrated the highest IRES activities of the 5′ UTR of the lineage 2 virus in cell lines, including neuronal and lung cells (Figure 3A). Mutations in stem-loop II, V, and VI are known to affect the neurovirulence of enteroviruses, including poliovirus [8,9,10], and stem-loop IV plays an important role in the IRES-mediated translation, possibly through binding to the host’s proteins [31,32,33]. We found that high IRES activity of the recently circulating lineage 2 strains is due to mutations in stem-loop IV (Figure 3B). Although recently circulating strains have shorter and different nucleotide sequences in variable regions in the 5′ UTR, compared with Fermon/1962, the difference did not affect the IRES activity. Further study is needed to elucidate which specific mutation in stem-loop IV is responsible for the increased IRES activity and the mechanisms of cell lines-dependent differences in IRES activities among strains.

Although we found no effect of the mutations in the 5′ UTR on viral growth (Figure 4), it is still possible that the increased IRES activity of recently circulating strains contributes to symptom severity and to the increased neuronal and/or respiratory pathogenicity. Differences in IRES activity can be reflected by altered levels in the production of polyprotein and altered levels of virulence factors, such as the viral 2A and 3C proteases. The effect of mutations in the 5′ UTR should be studied in the future possibly using in vivo model because evolution of the 5′ UTR may have, to some extent, played a role in the recent surge of this virus and/or its symptoms.

## Supplementary Materials

The following are available online at https://www.mdpi.com/1999-4915/11/7/626/s1, Data S1: Genomic sequence of 374 strains of EV-D68, Figure S1: RNA secondary structure of the 5′ UTR of EV-D68.
