# Supplementary material for: Evolutionary and Functional Diversity of the 5′ Untranslated Region of Enterovirus D68: Increased Activity of the Internal Ribosome Entry Site of Viral Strains during the 2010s
Source: Viruses. 2019 Jul 8;11(7):626. doi: 10.3390/v11070626 (PMC6669567; doi:10.3390/v11070626)
Supplement: Supplementary file 1 [file viruses-11-00626-s001.zip › figureS1 to proofreading.pdf]

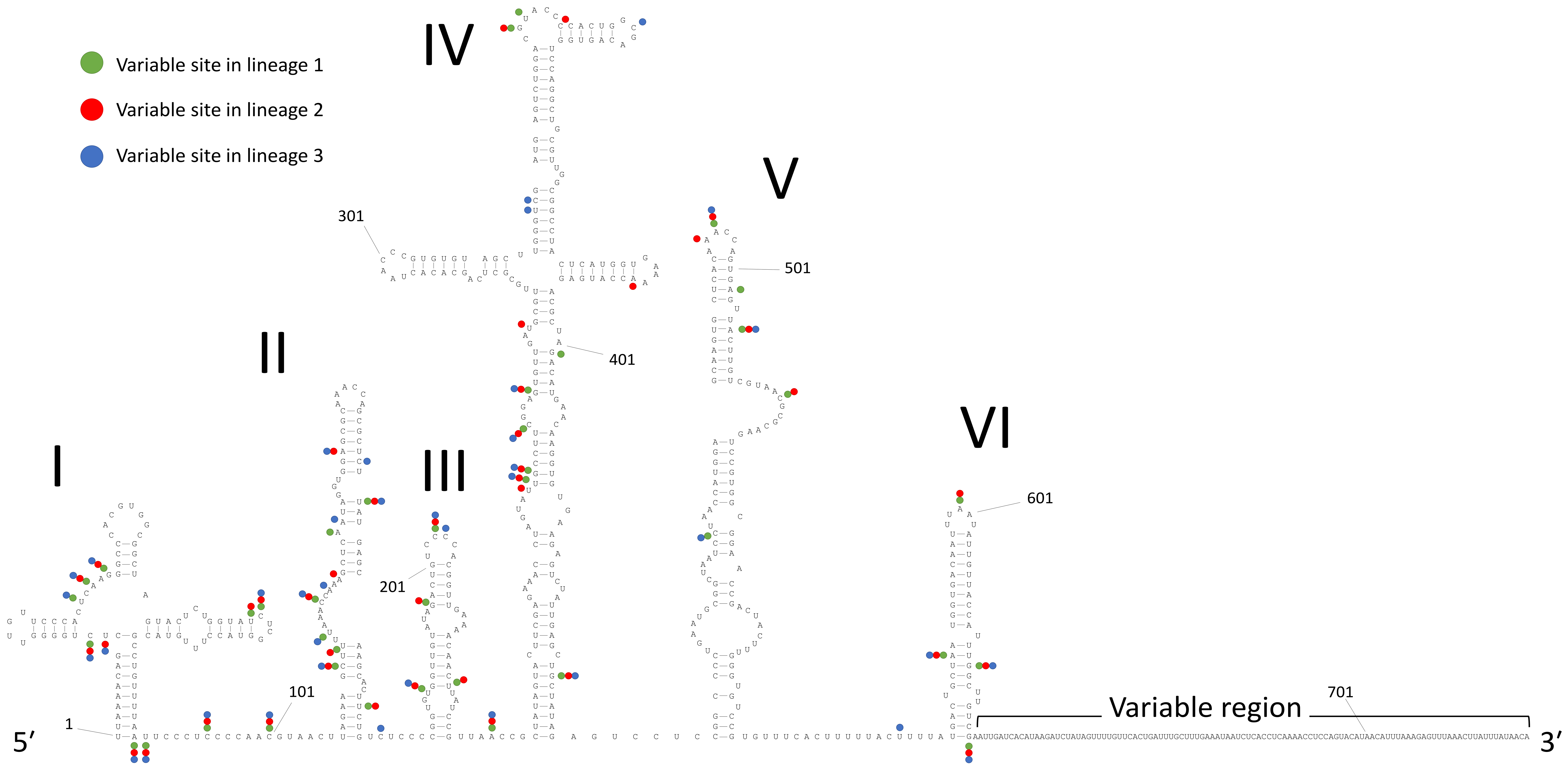

Figure S1. RNA secondary structure of the 5′ UTR of EV-D68

Predicted RNA secondary structure of the 5′ UTR of EV-D68 (Fermon/1962). Locations of mutations in the consensus sequence of viral strains in each phylogenetic lineage are indicated by dots except variable region. The Green, blue, and red indicate mutation sites in lineages 1, 2, and 3, respectively. This is the high-resolution version of Figure 2.
